# Supplementary material for: Loss of MGA repression mediated by an atypical polycomb complex promotes tumor progression and invasiveness
Source: eLife. 2021 Jul 8;10:e64212. doi: 10.7554/eLife.64212 (PMC8266391; doi:10.7554/eLife.64212)
Supplement: Supplementary file 2. — (a) Real-time mouse primers used in the study. (b) Real-time human primers used in this study. [file elife-64212-supp2.docx]

Supplementary File 2a: Real time primers (mouse) used in the study

| **Gene** | **Forward** | **Reverse** |
| --- | --- | --- |
| Rpl4 (housekeeping) | CCGTCCCCTCATATCGGTGTA | GCATAGGGCTGTCTGTTGTTTTT |
| Podxl2 | CCCACTCAAGATGTCCTTTCC | TCACCAGCACTACAAAGAGC |
| Gpc2 | TCTTACTCGGCTTACTTCAACTG | GCTTCGCTGACCACATTTC |
| Stag3 | TCCAGTTGAGTCTGCACAAAG | CCCCTCTATGTCCTCTTGATTC |
| Snai1 | GTGAAGAGATACCAGTGCCAG | AAG ATG CCA GCG AGG ATG |
| Itga1 | GACAGCCCTTGGAATAGACAC | CTC ACA GTC TTG GAT GAC CTG |
| Snai2 | ACACATTAGAACTCACACTGGG | TGG AGA AGG TTT TGG AGC AG |
| Tdrd1 | TTCTGCTCTGTCAAGGTCATG | TTC ACT CCA TAC CCC ATT TCC |

Supplementary File 2b: Real time primers (human) used in the study

| **Gene** | **Forward** | **Reverse** |
| --- | --- | --- |
| GUSB  (housekeeping) | CCTGCGTGTCCCTTCCTC | CGTTCTGGTCTGCCGTGAA |
| PODXL2 | CCCAGCGAAGAGAATGAAGAG | AATGGAACCTGCCTTCTCAG |
| STAG3 | GAGTGGTACATCGTCATAGCC | AATCCTGCATCCTGGTCTTG |
