# Supplementary material for: Loss of MGA repression mediated by an atypical polycomb complex promotes tumor progression and invasiveness
Source: eLife. 2021 Jul 8;10:e64212. doi: 10.7554/eLife.64212 (PMC8266391; doi:10.7554/eLife.64212)
Supplement: Transparent reporting form [file elife-64212-transrepform.docx]

***eLife’s* transparent reporting form**

We encourage authors to provide detailed information *within their submission* to facilitate the interpretation and replication of experiments. Authors can upload supporting documentation to indicate the use of appropriate reporting guidelines for health-related research (see [EQUATOR Network](http://www.equator-network.org/%20)), life science research (see the [BioSharing Information Resource](https://biosharing.org/)), or the [ARRIVE guidelines](http://www.plosbiology.org/article/info:doi/10.1371/journal.pbio.1000412) for reporting work involving animal research. Where applicable, authors should refer to any relevant reporting standards documents in this form.

If you have any questions, please consult our Journal Policies and/or contact us: [editorial@elifesciences.org](mailto:editorial@elifesciences.org).

**Sample-size estimation**

- You should state whether an appropriate sample size was computed when the study was being designed
- You should state the statistical method of sample size computation and any required assumptions
- If no explicit power analysis was used, you should describe how you decided what sample (replicate) size (number) to use

Please outline where this information can be found within the submission (e.g., sections or figure legends), or explain why this information doesn’t apply to your submission:

A minimum sample size of 3 per group was used for all experiments. Power analysis wasn’t required for in vitro experiments as results were fairly consistent between groups. Sample size information has been included in figure legends.

For in vivo studies, power analysis was performed as part of IACUC protocol preparation.

**Replicates**

- You should report how often each experiment was performed
- You should include a definition of biological versus technical replication
- The data obtained should be provided and sufficient information should be provided to indicate the number of independent biological and/or technical replicates
- If you encountered any outliers, you should describe how these were handled
- Criteria for exclusion/inclusion of data should be clearly stated
- High-throughput sequence data should be uploaded before submission, with a private link for reviewers provided (these are available from both GEO and ArrayExpress)

Please outline where this information can be found within the submission (e.g., sections or figure legends), or explain why this information doesn’t apply to your submission:

Information on number of independent experiments and biological replicates are detailed in figure legends. Technical replicates weren’t counted towards sample size for any experiment. Graphs have been plotted showing individual datapoints for almost all figures and outliers haven’t been excluded. High throughput sequence data has been submitted to GEO. The accession numbers are as follows: mouse Kras tumor RNA Seq (GSE161609), mouse KP cell line RNA Seq (GSE161606), Human colon organoid RNA Seq (GSE161543), mouse KP cell line ChIP Seq (GSE161541), human A549 CUT&RUN (GSE161539)

**Statistical reporting**

- Statistical analysis methods should be described and justified
- Raw data should be presented in figures whenever informative to do so (typically when N per group is less than 10)
- For each experiment, you should identify the statistical tests used, exact values of N, definitions of center, methods of multiple test correction, and dispersion and precision measures (e.g., mean, median, SD, SEM, confidence intervals; and, for the major substantive results, a measure of effect size (e.g., Pearson's r, Cohen's d)
- Report exact p-values wherever possible alongside the summary statistics and 95% confidence intervals. These should be reported for all key questions and not only when the p-value is less than 0.05.

Please outline where this information can be found within the submission (e.g., sections or figure legends), or explain why this information doesn’t apply to your submission:

Figure legends indicate statistical tests that have been used. When Student’s t-test hasn’t been used e.g. for in vivo studies the appropriate statistical test has been performed e.g. Welch’s t-test assuming unequal variance between groups. Mean, SEM and individual data points are clearly visible on all graphs. Exact values of N have been included along with exact p-values wherever applicable (in figure legends).

(For large datasets, or papers with a very large number of statistical tests, you may upload a single table file with tests, Ns, etc., with reference to sections in the manuscript.)

**Group allocation**

- Indicate how samples were allocated into experimental groups (in the case of clinical studies, please specify allocation to treatment method); if randomization was used, please also state if restricted randomization was applied
- Indicate if masking was used during group allocation, data collection and/or data analysis

Please outline where this information can be found within the submission (e.g., sections or figure legends), or explain why this information doesn’t apply to your submission:

For mouse studies, samples were allocated according to the genotype of tumors e.g. Empty vs. sgMGA treated.

For in vitro studies, groups were based on treatment e.g. control siRNA vs. siRNA of interest. Data masking was not utilized.

**Additional data files (“source data”)**

- We encourage you to upload relevant additional data files, such as numerical data that are represented as a graph in a figure, or as a summary table
- Where provided, these should be in the most useful format, and they can be uploaded as “Source data” files linked to a main figure or table
- Include model definition files including the full list of parameters used
- Include code used for data analysis (e.g., R, MatLab)
- Avoid stating that data files are “available upon request”

Please indicate the figures or tables for which source data files have been provided:

Source data for Enrichr analysis (ChIP-Seq data, CUT&RUN data) and GSEA (RNA Seq data) has been provided in supplemental tables 1-5.
